# Harnessing the benefits of yoga for myositis, muscle dystrophies, and other musculoskeletal disorders

**DOI:** 10.1007/s10067-022-06280-2

**Published:** 2022-07-19

**Authors:** Ahmad Saud, Maryam Abbasi, Holly Merris, Pranav Parth, Xaviar Michael Jones, Rohit Aggarwal, Latika Gupta

**Affiliations:** 1 Department of Medicine, Royal College of Surgeons Ireland, Dublin, Ireland; 2 Dubai Medical University, Dubai, United Arab Emirates; 3 Department of Rheumatology, Royal Wolverhampton Hospitals NHS Trust, Wolverhampton, WV10 0QP UK; 4 North Delhi Municipal Corporation Medical College, Delhi, India; 5 Cedars-Sinai Medical Center, Smidt Heart Institute, Los Angeles, CA USA; 6 Department of Medicine, University of Pittsburgh, Pittsburgh, PA USA; 7 City Hospital, Sandwell and West Birmingham Hospitals NHS Trust, Birmingham, UK; 8 Division of Musculoskeletal and Dermatological Sciences, School of Biological Sciences, Centre for Musculoskeletal Research, The University of Manchester, Manchester, UK

**Keywords:** Dermatomyositis, Inclusion body myositis, Inflammation, Muscular dystrophy, Myositis, Yoga

## Abstract

The recent global increase in popularity of home-based yoga, an ancient Indian technique practiced for thousands of years, has translated into its use as a complementary therapy for a multitude of ailments. This review aims to examine the published literature regarding the effects of yoga therapy on systemic chronic diseases; in particular on the inflammatory myopathies (IMs) and other muscle disorders.

Despite the fact that the evidence base for yoga in inflammatory myositis is in its infancy, collateral results in other disorders such as muscular dystrophies are promising. A beneficial effect of yoga in chronic pain has been shown alongside an improvement in motor function and muscle strength. Patients with Duchenne muscular dystrophy with respiratory involvement may find improvement in lung function. Elderly patients may experience reduction in falls secondary to an improvement in balance while practicing long-term yoga therapy.

Further benefits are improving disorders of mental health such as depression and anxiety. A reported improvement in overall quality of life further suggests its efficacy in reducing morbidity in patients with chronic diseases, who often suffer co-existent psychological comorbidities.

## Introduction

Yoga is a 5000-year-old ancient Indian practice designed to achieve serenity of the mind, attunement of the body and cleansing of the spirit [1, 2]. It is composed of a wide variety of techniques that integrate and harmonize the mind, body, and soul [3]. *Asanas* are a series of postures that intertwine with *Pranayamas* — controlled breathing techniques — through which psychological healing and self-realization are attained [1]. The harmonization between respiratory mechanics and the musculoskeletal system holistically challenges and optimizes body functioning by maintaining stillness and steadiness as the mind is allowed to achieve emotional mastery and a state of spirituality [1, 4]. Physiological mechanisms such as those facilitated by dopamine-beta-hydroxylase, monoamine oxidase, or adrenal steroids cause reduced autonomic activity resulting in a parasympathodominant state, thus soothing the body [5]. In doing so, yoga becomes a potential tool not only for the treatment of physical musculoskeletal disorders, but also ailments of the heart, lungs, and mind.

Research on the benefits of yoga has been expedited over the last decade as its increased popularity and uptake has become a global phenomenon. As more people find benefit in this ancient technique, it is inevitable that further focused research on yoga may materialize. Current data on the role of yoga in management of chronic diseases remains limited however. The purpose of this article is to explore the current evidence for yoga, and how it may pertain to Idiopathic Inflammatory Myopathies (IIM), muscle dystrophies like Duchenne and Becker muscular dystrophy, and systemic involvement often associated with IIM such as Interstitial Lung Disease (ILD), arthritis, and cardiovascular health.

IIM can begin as inflammation of the muscle and skin, but progression may lead to concurrent involvement of the respiratory and, rarely cardiovascular system as well [6]. Corticosteroids including prednisolone are often used as first line treatment in many subsets of IIM [7]. While corticosteroids are often crucial in management, they are seen as double-edged sword due to the multitude of short and long-term side-effects that may ensue [8, 9]. Therefore, complementary treatment methods are highly sought after for IIM [5].

The COVID-19 era has increased morbidity and reduced access to routine healthcare, to the detriment of many musculoskeletal diseases [10]. After medication, maintaining adequate physical activity is crucial for maintaining physical functioning in these patients [11]. Traditionally, exercise — often by means of physiotherapy — has been a beneficial adjunctive management option in treating the disease in these patients. Exercise therapies including yoga may be useful in patients with these debilitating illnesses due to their effects on muscle strength and endurance [12]. The difficulties in the procurement of drugs and adherence to in-person physiotherapy sessions have left a void in the ongoing care of patients suffering from these debilitating diseases in the last 2 years [10]. This brings into focus the need for alternative and ancillary treatments in the long-term management of these patients [10, 13].

Due to its ease of use as a home-based therapy, yoga may be an option in the management of patients with these myopathies. Yoga is a practice believed to holistically affect both the mind and body which may be a useful adjunctive measure in targeting the ailment at its root cause rather than treating the disease ex post facto.

## Methods

**Fig. 1:**
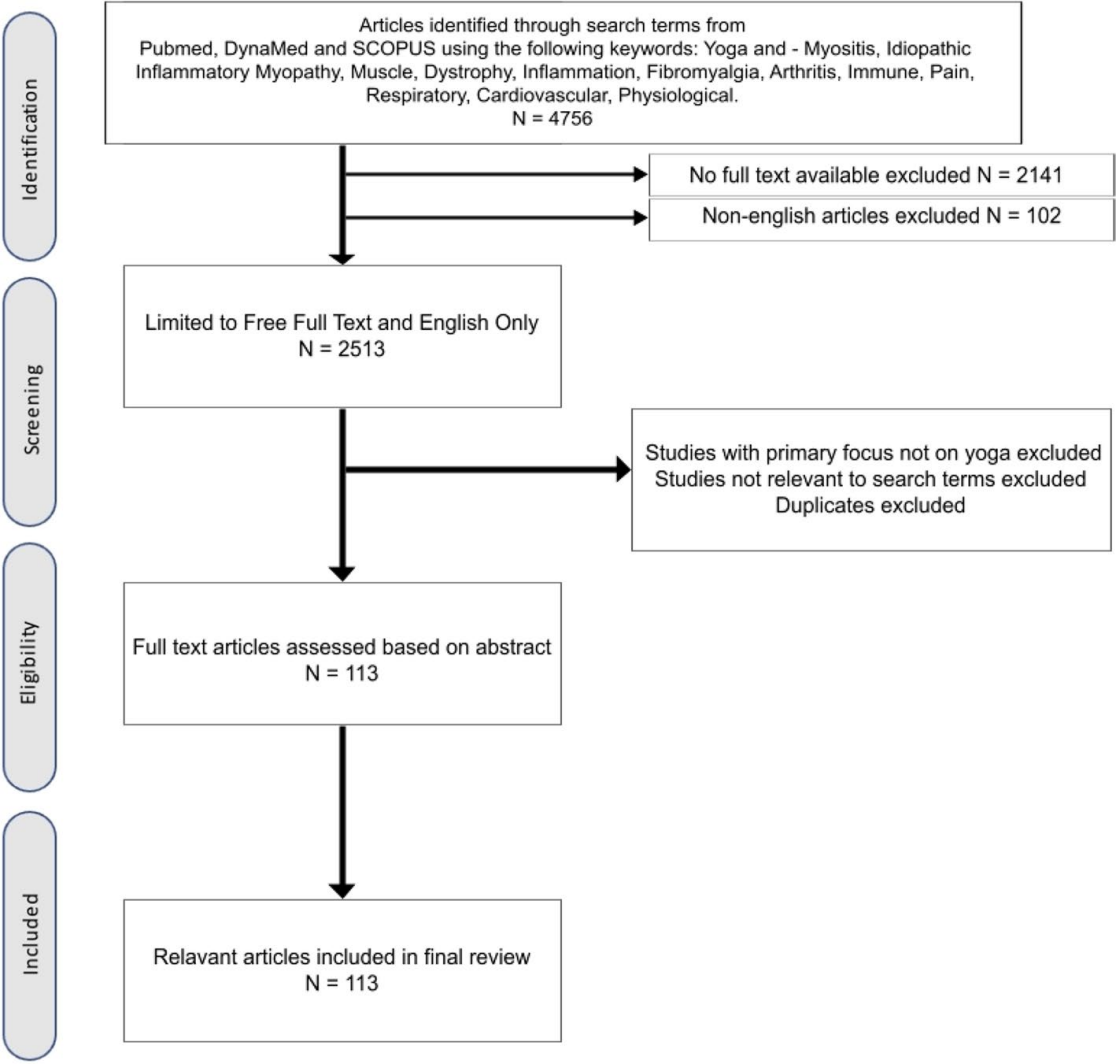
Flowchart of study selection processusing Pubmed, DynaMed and Scopus databases

In July 2021, we conducted searches on MEDLINE and Scopus using the search terms Yoga AND Myositis and Yoga AND Muscle using the strategy previously described by Gasparyan et al. to find 2 and 5 articles on the effect of yoga on Inflammatory myositis and muscular dystrophies, respectively [14]. Of these, one article on Inflammatory Myositis and four on Muscular dystrophies were shortlisted for exploration. We further explored other evidence-based studies obtained from the DYNAMED database and google scholar for evidence of collateral benefit of Yoga on the cardiovascular and musculoskeletal system, and mental health, in chronic conditions to analyze and discuss potential benefits in patients with muscle diseases in this narrative review. The search terms used are detailed in Fig. 1.

## Yoga and musculature

**Fig. 2:**
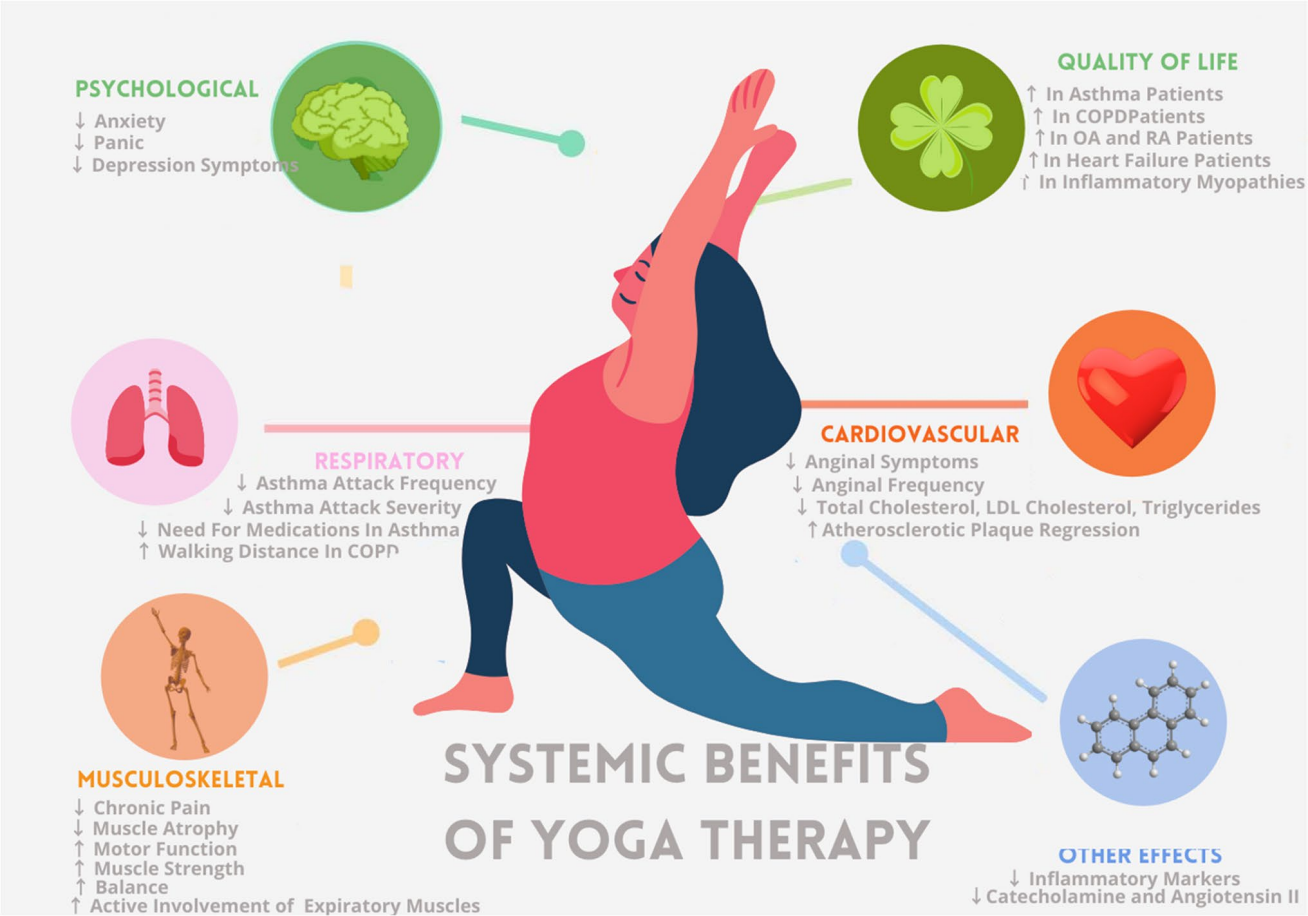
An infographic depicting the positive impact of yoga therapy on the cardiovascular, respiratory, and musculoskeletal system, along with the psychological impact, and effect on patient Quality of Life (QoL) in chronic health conditions

The pleiotropic effects of yoga on the heart, lungs, vasculature, and cognition, as shown in Fig. 2, have previously been discussed in the literature. Current studies on the effects of yoga on the musculature however are scarce, and accurate interpretation is limited by small sample sizes.

Available studies report that benefits of yoga on the musculature may include, but are not limited to, enhancement in muscle torque, improvement in hand grip strength and muscle dexterity, decreased lower back pain, delay in onset of muscle soreness, as well as increased flexibility and balance [15–20]. Studies assessing effects of yoga on IIM and muscular dystrophies are recently starting to emerge, although more conclusive findings with larger number of participants are required before the efficacy of yoga can be substantiated in these diseases.

### Muscle recruitment in yoga therapy

Previous studies have shown positive benefits of yoga on muscle strength and flexibility (Fig. 2). *Asanas* strengthen the back and relieve backache by alleviating muscular spasms, increasing flexibility, and decreasing body weight [20–22].

A yoga breathing workout-session comprising of twenty breaths can be considered equivalent to the work required to perform one hundred conventional abdominal crunches [17]. This is due to the heavy recruitment of the rectus abdomini and external oblique muscles during yoga practice. Furthermore, studies comparing yoga breathing exercises to abdominal crunches found more abdominal muscle involvement during yoga breathing exercises (41% muscle activity) when compared to standard abdominal crunches (24% muscle activity as determined by means of electromyogram (EMG) studies). The oblique muscles were also found to be more effectively involved in yoga than other exercise therapies [17]. 

In the elderly, muscle strength activities such as yoga may help in counteracting age-related muscle loss (sarcopenia), enhancing functional performance, improving bone mineral density (BMD), and decreasing the number of falls [4, 12, 23].

### Effects of yoga in idiopathic inflammatory myopathies (IIMs)

IIMs and muscular dystrophies like Duchenne Muscular Dystrophy (DMD) primarily affect the musculoskeletal system, with over 80% of IIM patients presenting with muscle involvement. Some forms of myositis like the anti-synthetase syndrome may present without muscle involvement, however these too involve the muscle in up to 50% of cases either at disease onset, or eventually on follow-up [24].

Muscle weakness leads to significant disability in activities of daily living and decreased quality of life. Even after treatment, patients may continue to experience persistent muscle weakness, fatigue or pain due to muscle damage from chronic inflammation. Furthermore, patients with respiratory and cardiac involvement or associated comorbidities may suffer additional debility.

To date, only one article has been published looking at the effects and benefits of yoga in patients with myositis (Table 1). In this study published in February 2021, Kong et al. examined the patients’ self-reported perception of difficulty in performing activities of daily living (ADLs), as well as muscle strength, both before and 8 weeks after weekly yoga intervention [25]. The measured outcomes were scores on a Myositis Activity Profile (MAP). This is a measure of the Activities of Daily Living (ADLs) and Manual Muscle Testing (MMT) — measuring muscle strength in the axial, proximal and distal muscle groups [25]. The MAP score increased from 66.67 (SD: 25.34) before beginning yoga intervention to 69.17 (SD: 30.71) upon completion of the 8-week yoga program. While the difference between pre- and post-MAP scores was not a statistically significant one, examination of the individual components of the ADLs showed significant improvements in patient’s personal care, self-hygiene, and domestic activities. Muscle strength also increased, from 217.25 (SD: 41.65) pre-intervention to 228.25 (SD: 23.99) out of a possible 260 points on the Kendall 0–260 scale post yoga intervention [25]. This muscle strength improvement was found in both the proximal and distal muscle groups, even though statistical significance could not be determined due to the small sample size. This sole study into the effects of yoga in patients with myositis is limited by its small sample size however, and while a lack of statistical significance does not necessarily mean a lack of clinical significance, more studies with larger sample sizes warrant consideration.

**Table 1:** Impact of therapeutic yoga in muscle disorders

Author, year and country	Objectives	Type of study	Participants (n =)	Disease	Methods	Benefits reported
**Kong et al** **(2021)** **USA**	Evaluate effect of yoga on self-reported difficulties performing activities of daily living and muscle strength	Cohort study	6	Polymyositis, Dermatomyositis	MMT, ADL, MAP	Increase in muscle strength in all categories of MMT (217 ± 41.65 vs 228.25 ± 23.99 post treatment), improvements in patients' ability to perform ADL in every category of MAP questionnaire (activities of moving around, personal care and hygiene, domestic activities) except for movement [27]
**Rodrigues et al** **(2014)** **Brazil**	Effect of yoga breathing exercises on respiratory function	Prospective study	26	Duchenne Muscular Dystrophy (DMD)	FEV_1_ and FVC via spirometry	Improvement in respiratory physiologic parameters, such as FVC (82.3% ± 18.6% at baseline vs. 90.3 ± 22.5% at 10 months later) and FEV_1_ (83.8% ± 16.6% at baseline vs. 90.1 ± 17.4% at 10 months later) [31]
**Pradnya et al** **(2019)** **India**	To evaluate the effect of yoga therapy to modulate HRV in DMD children	Prospective study	124	Duchenne Muscular Dystrophy (DMD)	Heart Rate variability	The use of yoga in association with physiotherapy showed noteworthy changes in HRV parameters, which were very obvious after 9 months: Mean NN HRV (600.7 ± 126.2 in dual treatment vs 637 ± 92.3 in physiotherapy only), SDNN (55.1 ± 32 in dual treatment vs 61.3 ± 49.9 in physiotherapy only), RMSSD (51.3 ± 38.4 in dual treatment vs 61 ± 68.6 in physiotherapy only [33]
**Dhargave et al** **(2021)** **India**	Identify the add-on effect of yoga over physiotherapy on pulmonary functions in children with DMD	Prospective study	124	Duchenne Muscular Dystrophy (DMD)	FEV_1_ and FVC via spirometry	In DMD + yoga group, FVC (*P* < 0.001) and MVt (*P* = 0.004) significantly improved from baseline up to 1 year, whereas MVV (*P* = 0.007) improved from baseline up to 9 months. Tidal volume did not demonstrate a significant difference after 1 year of intervention in groups (*P* = 0.448 and 0.956, respectively) The improvements were steady and more pronounced in younger children [38]

Even though evidence is rather limited at the moment, yoga presents itself as a potential complementary adjuvant therapy in myositis patients alongside traditional pharmacological immunosuppression or in those in whom side effects of medication are unbearable. Yoga can therefore, be a cheap, effective and non-pharmacological adjuvant option for patients with IIM in resource poor settings. The distinct advantage of yoga in IIM lies in it being easy to practice, well tolerated and not requiring excessive in-person guidance, thus combating disability in IIM by easily maintaining routine physical activity [26]. The fear of damage or progressing symptoms resulting from exercise is a misconception, as lack of physical activity may spur muscle atrophy in myopathies. Furthermore, studies show that muscle weakness may persist even after long-term treatment with pharmacological therapeutic regimens [25].

### Effects of yoga in muscular dystrophies

Muscular dystrophies are another group of incurable muscle disorders and include Duchenne and Becker muscular dystrophy [27]. Yoga practice as an adjunct to medication has given promising results in muscular dystrophy, with significant improvement in respiratory function and heart rate variability, as is highlighted in Table 1 [28–31]. Performing *Sakthivikasaka* (a yoga practice aimed to improve overall muscular function) is considered equivalent to completing a session of moderate-intensity exercise [31]. *Sakthivikasaka* not only helps increase flexibility but also improves muscle strength, tone, and joint stiffness in patients [31].

Several mechanisms may account for the proposed beneficial effect of yoga interventions in muscular disorders. These may include a reduction in Catecholamine and Angiotensin II levels [32, 33]. An improvement in the bioavailability of nitric oxide is also a proposed mechanism as this has been shown to decrease blood pressure and improve blood supply to muscles [32]. These mechanisms are not only beneficial in muscular dystrophies but may be of crucial importance in patients with diabetic foot disease, in whom peripheral perfusion is impaired. This is an area that should undergo further studies which could help develop new guidelines and modify current ones [34].

## Yoga and the skeletal system

### Yoga effects on the joints

While the effects of yoga on myositis-associated arthritis have not specifically been studied, collateral evidence in other arthritides suggests a need for exploration of this domain. Yoga-related benefits in chronic inflammatory diseases like rheumatoid arthritis have been ascribed to decreased pain related disability [35]. In patients with rheumatoid arthritis, decreased ESR levels and improved disease activity scores have been described, though the study did not compare the effects of yoga to other exercises, only to a control group on medication [22]. Bussing et al.’s study examined several randomized controlled trials on yoga intervention in rheumatoid arthritis, finding a consensus among all studies of a reduction of arthritis pain after yoga intervention [35]. Other studies have also reported a moderate sized effect on overall pain improvement which suggests that yoga can be a useful intervention for managing long-term pain in these chronic inflammatory diseases [36, 37].

Patients with knee osteoarthritis or rheumatoid arthritis further benefit from improved 6-min walking capacity, self-perceived quality of life and reduction in depression when participating in twice-weekly yoga classes [38]. For patients with osteoarthritis, The Osteoarthritis Research Society International (OARSI) makes a direct and strong recommendation (Table 2) that patients be enrolled in mind and body programs like yoga [39]. However, the data supporting the role of yoga in osteoarthritis is of only moderate quality, and thus the need for further larger studies in order to obtain higher quality evidence still remains [40].

**Table 2:** List of guidelines from 2009 to 2021 recommending the use of yoga as management for different systemic diseases, strength of recommendation and the quality of evidence available

Guideline Name	Condition	Recommendation	Level of recommendation	Quality of evidence	Year
**American College of Physicians Guidelines** [[37]	Chronic Low Back Pain	Gives a recommendation for offering non-pharmacological therapy such as yoga therapy as initial therapy for chronic low back pain	Strong recommendation	Low Quality of Evidence	2017
**EULAR** [56]	Fibromyalgia	Meditative movements including tai chi and yoga may improve symptoms	Weak Recommendation	Low Level Evidence	2017
**American Diabetes Association (ADA) **[80]	Improving Physical Activity	In older adults with diabetes, physical activity in the form of yoga may be used to help increase flexibility, muscle strength, and balance	ADA Recommendation Grade C	Low Level Evidence	2021
**Osteoarthritis Research Society International (OARSI) **[39]	Knee Osteoarthritis	Structured land-based exercise programs involving the mind and body such as yoga therapy can be used with or without dietary weight management in adults with knee osteoarthritis	Strong Recommendation	Moderate Level Evidence	2019
**Canadian Network for Mood and Anxiety Treatments (CANMAT) guidelines** [85]	Depression	Yoga can be considered as second-line adjunctive treatment for the management of mild-to-moderate depression	Second line treatment option	Moderate Level Evidence	2009
**American Heart Association** [78]	Hypertension	Consider online yoga, dance, exercise classes	NA	NA	2021
**2021 GINA Report, Global Strategy for Asthma Management and Prevention** [74]	Asthma	Yoga, breathing exercises, biofeedback and aerobic training can be considered alternative asthma treatments	NA	NA	2021

### Stability of bone loss and fracture risk

Increased vertebral fracture risk has previously been described in IIM patients independent of postmenopausal status and glucocorticoid use [41]. These vertebral fractures are often asymptomatic until the late stages of the disease and are often exacerbated by immobility which can further increase fracture risk [41]. The vertebral fractures can result in a vicious cycle of future fractures owing to inhibition of osteocyte conversion to osteoblasts [42].

The fracture rate in patients with IIM is reported to be about 26.2 per 100 patient years [42]. This is surprisingly greater than the fracture rate observed in other rheumatological conditions such as SLE and post-menopausal rheumatoid which have a fracture rate of 3.5 to 3.7 per 100 patient years. Furthermore, vertebral fractures in myositis often occur at a younger age of onset than would be expected as a result of simple age-related bone loss, with the average age of first fracture being 38 years [42].

As these patients are also prone to a higher fall risk, it is imperative that bone health be maintained from an early age. Yoga therapy has been shown to help maintain stability of bone mineral density in post-menopausal women, effectively preventing decline in bone strength [43]. Furthermore, yoga therapy has been shown to decrease pain levels and improve patient functioning which can improve mobility in these patients [36]. It remains to be seen however, whether the beneficial effects of yoga on maintaining bone density and strength observed in post-menopausal women can extend to patients with IIM, especially for the axial muscles. If so, yoga therapy could be beneficial in reducing fracture risk in patients with IIM and this may be a worthwhile avenue for future research.

## Effects on the immune system

IIMs are largely autoimmune conditions and hence appropriate anti-inflammatory modulation is critical in the long-term management of these patients. Studies on the potential anti-inflammatory effects of yoga therapy are being reported in the literature.

Recent studies have shown that regular and intensive exercise promotes an anti-inflammatory state while promoting muscle growth. This has been hypothesized to be due to downregulation of pro-inflammatory genes and the immune response [3, 11]. Although the exact mechanisms behind the anti-inflammatory properties of yoga remain to be described, several studies have shown that patients who routinely practice yoga have lower concentrations of IL-6 and C-Reactive Protein (CRP) when compared with controls [44, 45].

Another study shows decreased amount of pro-inflammatory and profibrotic mRNA and an increased amount of anti-inflammatory and anti-fibrotic mRNA in yoga practitioners [46]. Furthermore, tissue fibrosis and extra skeletal muscle activity were both decreased after training, suggesting a protective effect of yoga in muscle damage and inflammation [47]. Studies of yoga trials in military personnel reported reduced exertion, attributed to more efficient oxidative stress management by increased glutathione metabolism, per-oxidation product elimination and antioxidant enzyme production [5]. The beneficial effects on oxidative stress have further been supported by other authors [22].

Eight weeks of yoga therapy was found to downregulate the transcription elements associated with cellular apoptosis, nuclear transport, metabolic processes, JAK-STAT cascade DNA replication, and importantly, T and B cell activation and chemokine signaling. In addition, there is an upregulation of DNA repair genes, and anti-inflammatory cytokines IL-2 and IL-4, IL-6 and TNF-α [22]. Several authors report concurrent findings [48–50]. While not specific to yoga, IL-6 release during exercise caused a rise in follistatin, aiding muscle growth and preventing skeletal muscle dysfunction, as well as DNA methylation, triggering structural and metabolic adaptations in skeletal muscles [51]. Chen et al. also noted a reduction in circulating CD41 + and CD-42b in yoga practice, though no increase in pro-inflammatory cytokines was reported after administration of TLR2 agonist. This suggests that yoga is associated with reduced TLR2 expression [49]. Further studies examining these findings are required, as standard therapeutic guidelines for IIM treatment are yet to be established, and pharmacological treatment has reported mixed results [52].

Literature generally supports the hypothesis that yoga decreases pro-inflammatory cytokines and increases anti-inflammatory cytokines, though other studies report increases in both inflammatory and non-inflammatory cytokines, or no difference at all [53, 54]. Decreased methylation of TNF regions in yoga practitioners has also been reported, though the functional impact requires further exploration [55]. Other studies showed significantly better disease activity when yoga was used as an adjunct to DMARDs compared with DMARD therapy alone, as well as upregulation of anti-inflammatory HLA-G [22].

These quantifiable anti-inflammatory effects of yoga suggest that there are definitive physiological mechanisms at work which could be harnessed in the treatment of inflammatory muscle conditions such as IIM.

## Benefits of yoga on pain

Conditions such as IIM and muscular dystrophies are often characterized primarily by muscle inflammation and weakness, but they may also present with substantial pain and tenderness. This can reduce quality of life, lead to reduced mobility and further amplify long-term sequalae such as sarcopenia and poor mental health. Yoga therapy may be of assistance in keeping patients physically active in long-term disease states, while catering for the mind as well as helping to improve pain [56].

Previous comprehensive metanalysis concluded that yoga improved motor function, reduced functional limitation and lowered pain in patients with mild-to-moderate lower back pain, fibromyalgia, and other rheumatologic conditions [36, 57]. Twelve-week yoga therapy, as advised by evidence-based expert recommendation, was also found to alleviate pain in Multiple Sclerosis [58]. Wieland et al. reported that yoga therapy significantly improved back pain levels compared to non-yoga controls such as stretching or regular exercise [37]. On a 0–24-point Roland-Morris Disability Questionnaires scale, these patients reported a 2.15 point [CI: 1.08–3.23] improvement in back-function after 6 months of yoga therapy, which can be classified as a small to moderate improvement in back function [37].

Other studies highlight benefits of yoga when used adjunctively in usual treatment regiments, reporting marked improvements in back-related disability in patients with chronic back pain practicing yoga compared to those undergoing usual care only [18].

Currently, the American College of Physicians recommends yoga as the first line non-pharmacological management for patients with chronic low back pain as seen in Table 2. This recommendation stems from the results of a Cochrane review finding that yoga improves daily functioning in adult patients with chronic back pain and decreases pain levels [37]. Twelve randomized controlled trials were included in the review, examining 108 adults who received hour-long weekly yoga sessions [37]. In studies comparing yoga treatment to a non-exercise control group, a mean difference of 7.81 points [CI: 2.25–13.37] (on a 100-point scale) was observed between the two groups after 6 months, suggesting that there is an improvement in self-proclaimed pain scores after yoga treatment [37].

As a reflection of the above, a study of electronic health records (EHR) at Penn Medicine between 2006 and 2016 found a tenfold increase in the prescribing of yoga as a treatment modality by primary care physicians for a range of musculoskeletal conditions such as lower back pain, myalgia and myositis [59]. This could signal an already increasing acceptance of yoga as a therapeutic intervention.

In the short term, it has been proposed that yoga could be utilized in a peri-operative setting [60]. The Center for Disease Control (CDC) recommends that non-opioid analgesia be attempted first. In the peri-operative setting interventions like yoga, meditation, physical therapy, or swimming therapy may be combined with non-opioid analgesia for synergistic pain relief.

Heightened pain perception can result in decreased quality of life for patients with IIM and other musculoskeletal pains [61]. Furthermore, factors like depression, anxiety and impaired sleep can have an overbearing effect on pain perception. There is, however a lack of studies examining the effects of exercise training routines on pain perception in IIM. Misse et al. conducted the first systematic review on this topic and reported a lack of significant improvement in pain perception after exercise training in IIM patients [61]. Due to the heterogeneity of the data, however, they recommended further research allowing for potential confounding factors such as poor mental health which are often comorbid in IIM.

### Effects of yoga in fibromyalgia

In contrast to the muscle weakness seen in myositis, fibromyalgia is instead distinguished by intense pain and stiffness. Compounding the morbidity in these chronic diseases are bowel and bladder disturbances, depression, anxiety, and restless sleep [33]. Currently, analgesic medications are used for symptom control, but adjuvant therapies in the form of exercise programs and regimens are much sought after, though studies on such modalities are scarce [62].

It is not surprising that strengthening the abdominal and lower back muscles by means of yoga therapy can help improve bowel and bladder function, and in those prone to falls such as the elderly, can decrease falls by maintaining balance [4, 23]. EULAR recommendations (Table 2) suggest that yoga may have a beneficial effect on symptom control in fibromyalgia patients. Other authors report that relaxation yoga can be efficacious in improving pain levels as well as promoting functional improvements in fibromyalgia patients [62, 63].

A randomized controlled trial by Serrat et al. of 169 fibromyalgia patients found that adjunct use of nature-based activities including yoga, was more effective than usual exercise therapies in improving patient mood, confidence, fatigue, pain, stress, and exercise capacity [64].

Disturbance in sleep and fatigue in fibromyalgia patients was found to improve with yoga therapy, with a 29.9% reduction in fatigue and 23.9% reduction in poor sleep [65]. Eight week yoga practice in fibromyalgia patients demonstrated reduction in anxiety by 42.2%, depression by 41.5%, and emotional distress by over 30.1% [65]. Yoga was shown to decrease pain catastrophizing by 16% on the Pain Catastrophizing Scale (PCS). The greatest pain reduction was observed in those practicing yoga for 25 min or more a day, over the 6-week period of the study [66]. Long-term studies with larger sample sizes, and further research on specific yoga *asanas* are still warranted for more evidence for the utility of yoga in fibromyalgia.

## Benefits of yoga on function in the elderly

In elderly cohorts, declining muscle function is a major contributor to falls risk and reduced ability to perform activities of daily living [67]. Meta-analyses examining the effects of yoga in the elderly population reported improved physical fitness, muscle strength, power, endurance, flexibility in those in their 60 s and 70 s [57, 67]. In a study of 7 trials with 1033 participants, bi-weekly 45 min yoga sessions showed a significant improvement in fatigue in the elderly, at 3 months post-intervention when compared to controls [SMD: − 0.40, 95% CI − 0.62 to − 0.18], though this effect did not persist long-term [68]. The most effective treatment regimen for improving physical fitness in this study required 9–12 weeks. However, the loss of benefits occurred rapidly if patients were inconsistent or practiced yoga for less than 9–12 weeks, while treatment regimens longer than 12 weeks caused a large number of participants to lose interest and decreased adherence to the yoga therapy program [68].

## Additional systemic benefits of yoga

IIM is an umbrella term that encapsulates autoimmune diseases like dermatomyositis, polymyositis, and inclusion body myositis. Cardiovascular, respiratory, and psychological disorders are often comorbid with these conditions, as elaborated below. Figure 2 summarizes the benefits of yoga therapy on various body systems, Table 3 summarizes the evidence of systemic benefits of yoga therapy, and Table 2 shows guidelines from 2009 to 2021 recommending the use of yoga as management in various systemic diseases [13, 28, 29, 31, 44, 70,71,72, 73, 74, 75,, 76, 77, 78, 79, 80, 81, 82, 83, 84, 85].

## Challenges and limitations in implementing yoga

A wider dissemination of accurate information to health-care practitioners is much needed before population-wide uptake of this modality can be observed, particularly in countries that are not so familiar with yoga therapy.

It must be noted however, that some studies do report adverse events like back pain or injuries following yoga practice [37, 84]. However, these may be attributable to inappropriate posturing or lack of guidance during therapy [1]. Most of the adverse events observed were from extreme postures such as head stands. While yoga therapy remains at least as safe or safer than traditional exercise treatments according to the study, it is crucial that the correct technique be taught to people, and that patients do not attempt postures that may be excessively uncomfortable for them in order to prevent potential injury.

## Conclusion

Yoga, even though not a proven treatment for the aforementioned muscle disorders, can be a useful complementary therapy without prominent side effects. Yoga may be particularly useful in palliative management and in improving the prognosis of musculoskeletal and non-musculoskeletal diseases [85]. Yoga can provide patients a sense of internal and external motivation by making them feel a sense of control and calmness within themselves, at a time when their disease may seem like a burdensome life-long problem.

IIM and muscular dystrophies are disorders for which a definitive cure does not yet exist. Immunosuppression commonly used as a treatment option for IIM presents as a double-edged sword due to the plethora of side effects. Hence, adjunctive and complementary non-pharmacological therapy for these diseases is much sought after. Expert opinion generally supports the use of yoga in the management of musculoskeletal, respiratory, and psychological disorders. Current studies however are limited by their small sample sizes.

New studies on yoga are constantly emerging which suggest that yoga is a treatment option that we will get to see much more of in the future. Based on the current evidence, it is also important for physicians to be aware of yoga as a potential complementary therapy in patients with cardiac, respiratory and psychological conditions, and that they stay informed on any new guidelines that may be released in the future.

## Declarations

### Patient and public involvement

Patients or the public were not involved in the design or conduct, or reporting, or dissemination plans of our research.
